# ADHD: Is There an App for That? A Suitability Assessment of Apps for the Parents of Children and Young People With ADHD

**DOI:** 10.2196/mhealth.7941

**Published:** 2017-10-13

**Authors:** Lauren Powell, Jack Parker, Valerie Harpin

**Affiliations:** ^1^ School of Health and Related Research University of Sheffield Sheffield United Kingdom; ^2^ Sheffield Children's Hospital NHS Foundation Trust Ryegate Children's Centre Sheffield United Kingdom

**Keywords:** attention deficit disorder with hyperactivity, mobile applications, technology

## Abstract

**Background:**

Attention-deficit hyperactivity disorder (ADHD) is a highly comorbid disorder that can impact significantly on the individual and their family. ADHD is managed via pharmacological and nonpharmacological interventions. Parents also gain support from parent support groups, which may include chat rooms, as well as face-to-face meetings. With the growth of technology use over recent years, parents have access to more resources that ever before. A number of mobile apps have been developed to help parents manage ADHD in their children and young people. Unfortunately many of these apps are not evidence-based, and little is known of their suitability for the parents or whether they are helpful in ADHD management.

**Objective:**

The aim of this study was to explore the (1) parents’ views of the suitability of the top ten listed apps for parents of children and young people with ADHD and (2) the views of clinicians that work with them on the suitability and value of the apps.

**Methods:**

The top 10 listed apps specifically targeted toward the parents of children and young people with ADHD were identified via the Google Play (n=5) and iTunes store (n=5). Interviews were then undertaken with 7 parents of children or young people with ADHD and 6 clinicians who specialize in working with this population to explore their opinions of the 10 apps identified and what they believe the key components are for apps to be suitable and valuable for this population.

**Results:**

Four themes emerged from clinician and parent interviews: (1) the importance of relating to the app, (2) apps that address ADHD-related difficulties, (3) how the apps can affect family relationships, and (4) apps as an educational tool. Two additional themes emerged from the clinician interviews alone: monitoring ADHD symptoms and that apps should be practical. Parents also identified an additional theme: the importance of the technology. Overall, the characteristics of the current top 10 listed apps did not appear to match well to the views of our sample.

**Conclusions:**

Findings suggest that these apps may not fully meet the complex needs of this parent population. Further research is required to explore the value of apps with this population and how they can be tailored to their very specific needs.

## Introduction

### ADHD in Children and Young People

Attention-deficit hyperactivity disorder (ADHD) is a neurodevelopmental disorder, characterized by three core symptoms: hyperactivity, impulsivity, and inattention, which has a profound impact on the individual and their family [1-3]. ADHD is highly comorbid [4-6] and has a prevalence of 3% to 5% of school-aged children worldwide [7]. Furthermore, 80% to 85% of these children continue to be impaired by their ADHD symptoms as adolescents [1,8-10] and 60% as adults [2]. The presence of ADHD also increases the risk of premature death [11-13]. When ADHD persists into adulthood, affected individuals are more likely to engage in criminality and substance abuse [14-16]. ADHD also places a large economic burden on society. In the United Kingdom, in 2010, it was estimated that ADHD in adolescents alone cost £670 million to the National Health Service (NHS), social care, and education resources [17]. The total annual cost of ADHD for children in the United States is estimated at US $38 to $72 billion [18]. ADHD in children and young people (YP) is also extremely challenging for parents to manage [3].

Globally, ADHD treatments include pharmacological and nonpharmacological interventions [19-21]. In less severe cases, nonpharmacological behavioral interventions such as psychoeducation programs, behavioral interventions, and cognitive behavioral therapy are used alone, and in more severe cases, it is recommended that both approaches are used in parallel [19].

When young people diagnosed with ADHD reach their adolescent years, they are less likely to take their ADHD medication reliably and are more likely to disengage from services [22]. This trend in young people is mirrored across other services such as diabetes [23] and mental health services [24-28]. This is problematic for a number of reasons including the negative effect ADHD symptoms can have on academic attainment [4,29], which can often result in the young people having to repeat a school year [30]. Adolescents are more likely to be expelled from school [31] and spend less time in education because of truancy than their peers [32]. All of these problems pose huge challenges for parents.

### The Role of Parents in Child and Adolescent ADHD Management

Parents are under tremendous strain when caring for a child or YP with ADHD [3]. It is for this reason that the National Institute for Health and Care Excellence [19] guidelines recommend that all parents of children and young people diagnosed with ADHD are invited to attend parent management groups where they can build up a repertoire of strategies to help them manage their child’s ADHD and help their child achieve in life and at school [19].

Emphasis has been placed on the importance of the supportive role of the parent in ADHD [3,33]. Young people with ADHD often underreport their ADHD-related difficulties compared with their parents [34]. Therefore it is important that they have the support of somebody such as a parent or guardian to assist with their difficulties, as the young people doesn’t always appreciate how much difficulty they are having. Indeed, it is often the case that the parents require support for themselves so that they can provide optimal support for their child with ADHD. Available parenting interventions for the management of ADHD in primary school–aged children include an eight-session parenting behavioral program [35], the New Forest Parenting Programme [36], 123 Magic [37], and the Triple P Positive Parenting Program [38]. These programs involve strategies that cover aspects such as the understanding of ADHD and the challenging behaviors associated with it, specific behavioral strategies that address parent-child interaction, the use of time-out strategies to reduce problematic behavior, how to manage child behavior in public, and school and maintenance issues. Parent groups provide vital support for parents of children with ADHD and provide the parent with strategies that they can apply to managing their child’s ADHD outside the group. However, many of them use paper-based resources which may not provide instant advice at a time where a parent needs it and management strategies will usually need reinforcement over time.

### The Role of Technology in ADHD Management

The increasing use and accessibility of the Internet, mobile phones, and mobile devices offers an additional way to support parents [39]. Technology could be used for parents to engage their child in daily activities, monitor their symptoms, or to gain further advice on how to manage their child’s or young person's ADHD. A recent qualitative study has demonstrated that parents, children, young people with ADHD, and health care professionals appreciate the potential of technology to help with the ongoing management and monitoring of a child’s ADHD [40].

There have been a number of apps developed for parents for this purpose, but thus far, there is little evidence to underpin them. Some of these apps are designed for the parent and child to use jointly to enable the parent to monitor their child’s ADHD symptoms, monitor medication compliance, give parenting advice for managing a child with ADHD, and also to help with daily routines such as getting dressed and going to bed. Evidence includes app development papers such as the WHAAM app that involves monitoring ADHD behavior in children and data sharing between parents, teachers, and health care professionals [41]. One study uses an app and a skin conductance sensor to measure parental stress by notifying the parent during stressful times to make them self-aware of their emotions. However, a number of false-positive alerts were apparent during this study [42]. Another study used a mobile app to help improve morning and bedtime routines. Parents reported decreased frustration levels in this study [43]. Nevertheless, these studies focus on the development of a single app and contain low sample sizes. This means we cannot apply these findings to a wider population.

Furthermore, app quality is not routinely monitored. Once the app has been bought, there is little feedback on usefulness. There is also minimal guidance available to demonstrate the reliability, validity [44], and suitability of available apps. For example, the British Standards Institution has developed a report outlining a code of practice recommended when developing a health app to help ensure it is fit for purpose [45]. This includes, and is not limited to, acknowledging and catering for the app’s target audience. Unfortunately, many apps do not adhere to such guidelines. A review of the NHS Choices apps library was undertaken in England, and three components were focused upon: (1) data protection act compliance, (2) efficacy evidence, and (3) relevance to British people. Many apps that were identified in this review did not have an evidence base, and it was shown that privacy and data security arrangements were often unsuitable [46]. The thin evidence base for apps also means that we know very little about what would make an app suitable for a parent of a child with ADHD.

This study proposes to identify apps aimed at the parents of children and young people with ADHD and to interview the parents and specialist clinicians who work with them to explore what could make an app suitable in terms of appearance, content, and functionality for the parents of children and young people with ADHD. This will provide those developing apps for this population with some key components to consider.

## Methods

This research involved the identification of the top 10 listed apps aimed at the parents and guardians of children and young people diagnosed with ADHD. Subsequently, the parents tested the apps, and they were then interviewed to ascertain their views on the apps and to explore what they believed the key components are for apps to be helpful for them. Clinicians who work with children and young people with ADHD and their parents were also interviewed to explore their insights into how to make apps successful for this parent population.

### Research Question

The research question was as follows: are the 10 top listed apps that are specifically designed and marketed for the parents of children and young people with ADHD suitable and what are the key components for apps to be suitable for this population?

### Search and Identification of Mobile Apps

In July 2016, a search of mobile apps in the Apple iTunes store and the Android Google Play store was conducted. These databases were selected because they displayed systematically organized app rankings defined by algorithms unique to each app store, commonly known as app store optimization (ASO) [47]. For Apple, the primary factor is number of downloads; however there are also many other secondary factors such as keywords and visuals [48]. Similarly, the Android database is filtered according to multiple criteria, including the volume of ratings, value of ratings, and download growth [49]. Although this presents a potential bias as apps are selected according to the database’s own ASO, it is to some extent unavoidable unless all of the search results are downloaded for testing [50], which was beyond the scope of this study. Apps for iPads and tablets were reviewed rather than phones, as apps are available on phone and tablet devices. For this study, apps available on tablets have been reviewed as they are easier to discuss with parents and clinicians.

The search term used was “ADHD.” This is because other search terms such as “ADD,” “children,” “Young people,” and “parents” did not provide different results to the “ADHD” searches. The term “ADHD” was searched in both iTunes and Google Play.

Preliminary screening was conducted based on app titles, full marketing description, and screenshots of the apps potentially relevant for inclusion. The first 5 listed apps that met the inclusion criteria from each app store were included, giving a total of 10 apps for study (Textboxes 1 and 2).

**Figure 1:**
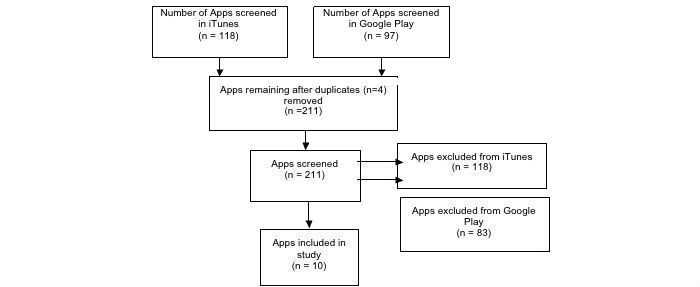
App selection process.

Duplicate apps were removed (Figure 1). This was applicable if there was more than one version of an app. It was decided that the app version to be included was the app that appeared first on the app store lists.

The remaining 10 apps were downloaded onto an Apple iPad Mini (Model: A1489) or a Samsung device (Model: GT-P5220), and their content was summarized by the lead author [18]. The apps were summarized into a tabular format to help assist participants during the semistructured interviews (Multimedia Appendix 1).

**Inclusion criteria of apps. (Please note that some selected apps were aimed at children and young people with attention-deficit hyperactivity disorder [ADHD], as well as the parents. These apps were included as long as they specifically stated they were for the parents as well. Apps were only excluded if the app was not intended for the parents in any way).**

Inclusion criteria

- An app that states it is aimed at a parent of a child or a young person with ADHD or attention deficit disorder
- Mobile app
- App is available in the English language

**Exclusion criteria of apps. (Please note that some selected apps were aimed at children and young people with attention-deficit hyperactivity disorder [ADHD], as well as the parents. These apps were included as long as they specifically stated they were for the parents as well. Apps were only excluded if the app was not intended for the parents in any way).**

Exclusion criteria

- Does not state app is aimed at parent of a child or young people with ADHD
- Not a mobile app (eg, magazine)
- Not available in the English language
- Duplicate app

### Participants

Convenience sampling was adopted to recruit specialist clinicians who work with children and young people with ADHD and their families, as well as parents of children and young people with ADHD. Parents were recruited via a Family Action group (a national charity that provides support for families) and an online ADHD community group. Clinicians were recruited via the NHS Child and Adolescent Mental Health Service in the South Yorkshire region. Participants were recruited until data saturation was achieved. Eligibility criteria for clinicians were that they had to be employed by a service that treats children and young people with ADHD.

Eligibility criteria for the parents of the children and young people with ADHD were as follows: (1) must be a parent of a young person who has a confirmed ADHD diagnosis and (2) must be able to provide details of the medication the child is prescribed.

### Procedure

Ten separate semistructured interviews took place in the Sheffield region. Three interviews with parents involved interviewing parents jointly. Interviews lasted up to 60 min with the clinicians, and up to 90 min with the parents. The study received ethical approval from the University of Sheffield’s School of Health and Related Research Ethics Committee (references 010768, 011377), as well as NHS Health Research Authority and Research and Development approval.

The researcher visited parents at the Family Action parent group and introduced the study. Parents then either contacted the researcher, or they requested that the researcher contacted them at a time convenient to them. Clinicians were approached via email. Interviews took place at a quiet convenient place with the clinicians and in the homes with the parents. Before the interviews, the clinicians and parents provided written informed consent.

Information about the study was sent to all participants at least 1 week before the interviews took place. At the beginning of each interview, the study was explained to the participants and questions were answered.

Clinicians and parents were presented with all 10 apps identified. Participants were given the opportunity to use the apps themselves during the interview. Interview discussions were guided by an interview schedule covering their views in four key areas:

- What makes a successful app?
- What makes an app less successful or unsuccessful?
- How could an app function benefit them as parents of young people with ADHD?
- How could apps for parents help manage ADHD or address difficulties in young people?

The two groups provided two unique perspectives: a user perspective and a clinician perspective. Participants also completed a short questionnaire on their demographic characteristics. Parents additionally provided details of their child’s diagnosis and prescribed ADHD medication (where applicable).

### Data Analysis

All interviews were audio-recorded and transcribed verbatim. Thematic analysis [51] was used to search for data patterns within and across the participant groups. LP and JP independently identified codes and themes from the transcripts. Discrepancies were resolved through group discussion in an iterative fashion between the authors. Themes identified aimed to capture the essence of the participant’s views.

**Table 1:** Summary of how app developers describe the features of their apps.

iTunes apps			Android apps		
App	App claims	How apps meet their claims	App	App claims	How apps meet their claims
1	News updates and research about ADHD^a^ and other conditions. Increases knowledge.	Condition-specific resource for news, features, and research	6	To help coordinate parents, caregivers, and teachers in the follow-up of children aged under 18 years with ADHD	Manage medication, plan daily activities, measure treatment results, self-assessment tools, and direct doctor and teacher communication via sister app
2	To change challenging daily routines into fun. Songs to help guide child to timely efficient task completion through consistency, repetition, rhythm, and rhyme.	Step-by-step directions of daily tasks through songs, monitoring chart, and coloring book reward	7	Addresses memory Provides information about ADHD	Memory games of different levels and ADHD key concepts quiz. Dialogues with a cartoon character. Links provided with ADHD information
3	Improves self-control, reduces hyperactivity, and improves attention, concentration, and focus.	Mindfulness Training	8	Monitor ADHD symptoms over time Download resources	Links to ADHD info or resources Sliders to record key times of day, charts, email charts, and appointment reminders
4	To help child improve dressing skills.	Learn to dress by dressing a cartoon character in order and imitate	9	Gain solutions to problem behaviors	Click on problem behavior and find a suggested solution and possible approaches. Related to special education
5	To help children and adults understand ADHD and how to manage it.	Interactive story about a boy and a character with ADHD	10	Create a visual schedule to support transition times during the daytime	User can illustrate sequence of tasks or substeps of a task and can label images with text

^a^ADHD: attention-deficit hyperactivity disorder.

**Table 2:** Demographic characteristics of parents.

Unique ID	Gender	Length of child’s ADHD^a^ diagnosis (years, months)	Child’s age (years)	Other diagnoses of child	Child medicated?
P1	F^b^	Undergoing ADHD assessment	11	ASD^c^	No
P2	F	4, 0	15	Not applicable	Yes
P3	F	0, 7	13	ASD and anxiety	Yes
P4	F	0, 7	13	ASD and anxiety	Yes
P5	M	1, 10	10	ASD	Yes
P6	M	0, 8	9	ASD	No; under assessment
P7	M	0, 8	9	ASD	No; under assessment

^a^ADHD: attention-deficit hyperactivity disorder.

^b^F: female.

^c^ASD: autism spectrum disorder.

**Table 3:** Demographic characteristics of clinicians demonstrating a total of more than 57 years of experience working with children and young people with attention-deficit hyperactivity disorder (ADHD).

Unique ID	Gender	Length of time working with population (years)	Current job title
C^a^1	F^b^	6	Consultant child and adolescent psychiatrist
C2	F	12	Consultant child and adolescent psychiatrist
C3	F	0.5	Trainee 2 psychiatry
C4	F	10	Primary mental health worker
C5	M^c^	20	Consultant child and adolescent psychiatrist
C6	M	13	Consultant child and adolescent psychiatrist

^a^C: clinician.

^b^F: female.

^c^M: male.

**Figure 2:**
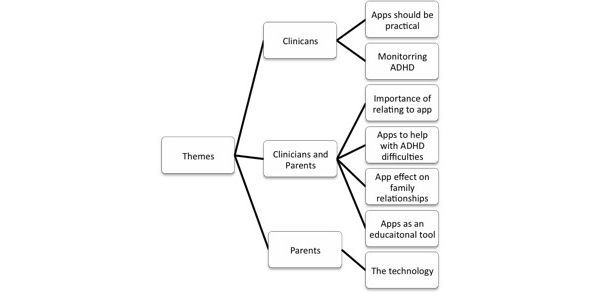
Themes identified during thematic analysis.

## Results

Five apps were identified from Google Play and five from iTunes in August 2016. Table 1 describes the claims the apps make within their individual descriptions and their contents.

### Participant Characteristics

A total of 13 participants were recruited from February to March 2017 (clinicians) and October to November 2016 (parents). Participants included 6 clinicians and 7 parents of young people diagnosed with ADHD. Participant characteristics are reported in Table 2 (parents) and Table 3 (clinicians). Two of the 7 parents were recruited via an online ADHD parent group in South Yorkshire. Five of the 7 parents were recruited from an ADHD parent group in Sheffield. Parents are only referred to this group if their child was diagnosed or being assessed for ADHD. All parents were able to provide information regarding the ADHD medication prescribed to their children, when applicable.

Data was collected until data saturation was achieved. Transcripts were available for all 13 interviews. During analysis, agreement between the 2 primary coders was high. Seven themes were identified in total (Figure 2). Where similarities between parents and clinician views emerged, from the data, they are combined and discussed under the same themes. These themes are identified, compared, and discussed below with illustrative quotations.

### Themes Identified by Parents and Clinicians

#### Importance of Relating to the App

Parents (n=7) and clinicians (n=4) believed it was important to relate to the app, especially when the app is aimed at both the parent and the child:

...if you could choose who you were, [referring to avatar in app] she [child] could relate a bit better.

P1

...that’s an issue already [referring to gender and race of apps character], it’s not even a boy is it, is it a girl or a boy?

P1

Clinicians (n=5) and parents (n=7) believed that apps should be visually attractive and have an appealing audio component (clinicians n=4; parents n=5):

I like quite visual things, I like visual things, I like stuff that takes 2 minutes, simple.

P1

Parents also believed that they should be able to alter apps to suit their own circumstances (n=7). Examples of this involve dressing a character in an order that is dictated by the parent; the parent could choose the items of clothing or the songs they play rather than the app dictating this. In terms of monitoring, they would like to choose the factors that they monitor rather than having these dictated by the app as well.

...having the option...at the beginning of being able to change the colors because he might go oh I can’t put that on because I don’t own a yellow t shirt...

P2

...like a [different] song every morning,...Michael Jackson, then Bob Marley, you know [tailoring the app to] what he [son] likes...if he woke up to something he likes it would put him in a better frame of mind [for the rest of the day]...

P7

#### Apps Should Target ADHD-Related Difficulties

Parents (n=7) and clinicians (n=6) also believed that the apps should target difficulties that specifically relate to ADHD, such as daily routines (clinicians n=3, parents n=7), behavior management (clinicians n=1), organizational skills (parents n=2), and to improve their communication with schools (clinicians n=2, parents n=2).

...if you’ve done that preparatory work [using an app to learn how to get dressed in the morning] with the child then hopefully that would make things easier [for the parents] in the mornings.

health care professional 2, HCP2

...they’ve got no organizational skills what so ever so that’s [app targeting organizational skills] really good.

P1

Two parents acknowledged that apps have the potential to reduce their ADHD-related anxieties (parents n=2). One clinician (HCP1) stated how an app could do this and why it could be beneficial:

...the parents’ reaction to the behavior...may be based on...day stress, my boss yelling at me so if I can do something to bring own my stress levels my response to the child with ADHD may be different.

HCP1

Almost all the participants (clinicians n=6, parents n=6) were able to highlight the benefits of using an app to monitor ADHD-related difficulties in ADHD:

...might spot trends as to why their behavior became the way it was.

P2

...to help you see where your kids struggling I would say.

P4

...good idea because...school can give more information...because I don’t see my child with the medication so it would be useful if I could see what the teachers are saying...?

P3

One parent believed that an app shouldn’t try to slow down a young person with ADHD but harness their hyperactivity by having an app that is also fast paced:

...having something that tries to slow em down just doesn’t work. You’re better off keeping them in one place by giving them something that will interact that’s fast.

P5

#### App Effect on Family Relationships

Parents and clinicians (clinicians n=3, parents n=3) noted that apps could be used to improve relationships between the parent and their child:

...it’s like more bonding time, education for myself and my son.

P6

I like the idea of parent and child working alongside [using an app].

HCP1

...bit of fun between the parent and child cos mornings and mealtimes and bedtimes can be really stressful so [the app] takes away some stress and introduces a bit of relationship building as well.

HCP4

It was also noted that apps could help address an ADHD diagnosis and what it means with siblings so that they can learn about their brother or sister’s condition (clinicians n=2, parents n=3):

...I think it would be useful to use with siblings [to explain ADHD to them] as well an’ they could interact with the game.

HCP2

#### Apps as an Educational Tool

Parents and clinicians liked the idea that apps could be used as an educational tool to learn about ADHD either via the app itself or via Web resources that are signposted within the apps (clinicians n=4, parents n=3). Parents also liked that some of the apps presented ADHD in a positive light (n=4):

...you can’t keep going on courses for the rest of your life. And you can’t always get it from a book as you either have to buy the book or the library doesn’t have it.

P5

...being reminded of positive things I also good for you and the kids self-esteem...

P3

Clinicians acknowledged that apps should be culturally relevant (n=2). For example, one American app provided news stories that provided information that isn’t always relevant to the United Kingdom:

...parents may be taking things on board that when you look into it there’s not much evidence for. Also, it’s promoting medications of therapies or whatever their whole healthcare systems structured differently to ours, which puts a bias on it...And also they have medication licenses that we don’t.

HCP2

One clinician stated that app developers should be mindful that parents also have varying abilities:

...it’s hard for parents to understand exactly what ADHD means...if you have a parent with...learning difficulties then its explained in an easy simple to understand way...

HCP2

### Themes Identified by Clinicians

#### Apps Should Be Practical

Many of the clinicians stated that parents of children and young people with ADHD lead busy and often hectic lives. Therefore, apps should be simple to use and easily integrated into their daily routines if they are to use them routinely (n=4). One clinician stated that if apps are visually appealing, then this would contribute to the ease of use of the product, which is important. They also believed that many parents may not want to pay for apps (n=3):

I don’t think the parents have got time for apps...I’ve met very few parents that will have time...most of them will have a sibling with ADHD or...younger children...

HCP3

...if you are if you’re a parent…trying to catch up on something in a fairly fast way,...I want something that will be quick...

HCP6

I wouldn’t spend that [as a parent].

HCP6

#### Apps to Monitor ADHD

Clinicians believed that apps could be a good tool to monitor the ADHD-related difficulties of a child, their symptoms relating to medication and also to provide reminders such as for appointments and taking ADHD medication (n=6):

...if they are able to keep that record [ADHD monitoring on an app] it may be helpful for the clinician who is monitoring the young person’s care...

HCP5

...the best thing is...it will remind you say at 9 o’clock at night and you can record the day.

HCP6; praising an app that reminds the parents to enter ADHD symptom information into the app on a daily basis

### Theme Identified by Parents

#### The Technology

Parents stressed the importance of the technology itself. They believed that an app isn’t always necessary (n=5); they can be distracting, and accessing the technology can sometimes be problematic (n=5). They also discussed how some of the apps required the input of identifiable information. Parents raised concerns here regarding data security (n=3);

...that’s all I do,...I type in ADHD in Twitter if I want to know about that I won’t download an app for it.

P1

Once again the only thing is it’s (the app) trapped in Apple world.

P4

...today your data might be safe, tomorrow your data maybe be going off on tour somewhere for a, for a price.

P4

Table 4 demonstrates the app characteristics identified by participants during semistructured interviews and which apps possessed these characteristics.

## Discussion

### Principal Findings

This study identified 10 apps that stated they were aimed at the parents of children and or young people with ADHD. Seven parents of young people with ADHD and 6 clinicians working with this population were interviewed. Interviews involved sharing the 10 identified apps with participants and asking what they think would make an app suitable for parents of children and young people with ADHD. Parents stated that technology access can be a barrier, that apps could enhance family relationships, apps have the potential to explain what ADHD means to effected siblings, apps should be flexible, and they could be used as an ADHD educational tool. Additionally, clinicians felt that apps would be useful for parents to monitor their child’s ADHD symptoms and related difficulties and that apps should be practical, quick, and easy to use to account for the parents with often busy and chaotic lifestyles. The latter findings are consistent with the findings of Simons et al [40].

As living with and managing the ADHD with a child or young person can be incredibly stressful for parents, the National Institute for Health and Care Excellence guidelines recommend that all the parents of children and young people diagnosed with ADHD should be invited to attend parent management and support groups [19]. There are a number of possible parent training or management programs available [35-38]; however, they cannot provide ongoing support, reminders, or instant advice at a time of crisis.

We are living in a society where technology is routinely used and embedded into our lives. Many apps are advertised for parents of children and young people with ADHD, but there is little evidence to underpin them and little evidence to ascertain what makes such apps suitable for parents. The young people and clinicians whose views were sought in this study did not find that the apps reviewed fully met their expectations. The highest match was 5 out of 8; two apps scored 1 out of 8, and the mean score was 3.6. Parents wanted the apps to be visually pleasing and tailored to their own circumstances. Clinicians were keen to highlight the possibility of using apps to allow parents to monitor their child or young person's ADHD-related difficulties. Clinicians saw the opportunity to use this data clinically.

**Table 4:** Summary of what makes an app suitable for a parent of a child or young person with attention-deficit hyperactivity disorder (ADHD), according to the parents and the clinicians interviewed in this study and which apps identified in this study include these characteristics. The apps have been scored out of eight. The scores represent how each app fits with the views of the participants.

Characteristics identified by participants as likely to be positive	iTunes						Google Play				
	App 1	App 2	App 3	App 4	App 5	App 6		App 7	App 8	App 9	App 10
Visually pleasing (ie, includes bright colors)	No	Yes	No	Yes	Yes	No		Yes	Yes	No	Yes
Allows personalization so that the user can relate to the app	No	No	No	No	No	Yes		No	Yes	No	Yes
Should help specifically with ADHD^a^-related difficulties	Aims to	Aims to	No	Aims to	Aims to	Aims to		Aims to	Aims to	No	No
Joint use (parent and child) to encourage health family relationships	No	Yes	Yes	Yes	Yes	No		Yes	No	No	Yes
Can be used as an educational tool or information source	Yes	No	No	No	Yes	No		Yes	No	Yes	No
App to monitor ADHD symptoms and difficulties	No	No	No	No	No	Yes		No	Yes	No	No
Should be practical (easy to use or embed into daily routine)	Yes	No	No	No	Yes	No		Yes	Yes	No	Yes
Attempts to improve daily routines	No	Yes	No	Yes	No	No		No	No	No	Yes
App score (out of 8)	3	4	1	4	5	3		5	5	1	5

^a^ADHD: attention-deficit hyperactivity disorder.

**Figure 3:**
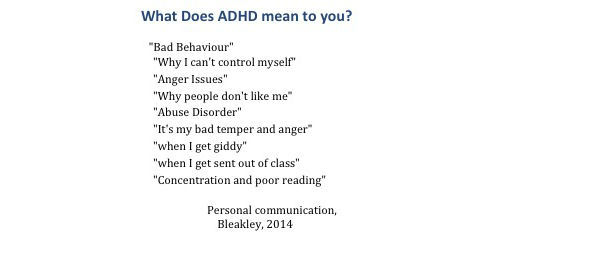
Replies from teenagers in an attention-deficit hyperactivity disorder (ADHD) clinic.

Increasing knowledge about a disorder, for example, by using psychoeducation, is recognized as important in improving management and then outcomes in chronic disorders. The knowledge of parents or carers of children with ADHD and, in particular, of young people with ADHD themselves, has been shown to be poor [47]. When young people in an ADHD clinic were asked what ADHD meant to them, they replied with negative answers that did not accurately represent the clinician’s views, as noted by Bleakley C (2014). A summary of these views can be found in Figure 3. Although young people valued direct information sharing with their doctor or nurse, they were also comfortable with using technology to improve their understanding [47].

The perspectives of both the clinicians and the parents are needed to build up a full picture. Clinicians are keen to make use of apps to promote positive behaviors and target specific needs. The views of the parents give guidance as to whether they would actually use the app and whether they felt it was likely to be helpful.

A Canadian study [52] considered parents’ attitudes to medical technology (genetic studies and magnetic resonance imaging scanning) and concluded that parents were in fact “In search of Anything that would help.” Indeed, their interviews reflected parents’ views that their needs were not being met despite increased understanding among health and education professionals. The choice of medications for ADHD has increased in recent years, but there is still a reluctance to use medication, and for many young people, achieving symptom improvement throughout their waking times, remains a challenge. Technology can now be accessed at any time in most places by most people, so it may offer the much needed extra support to young people and their families.

If technology is to be reliably helpful, some quality standards must be met. Apps are often developed rapidly with little evidence base. The pressure is often for financial success rather than proven benefit. This study gives us some insight into the quality of currently available apps and suggests that more research is needed to harness technology to benefit young people with ADHD and their families.

### Conclusions

This research suggests that currently the top 10 listed apps marketed for parents with young people with ADHD do not seem likely to meet their needs and crucially, do not have the key components of they believe will make an app helpful.

According to our participants, key components of a successful app are likely to be; pleasing visuals, the facility to personalize, the ability to help with specific ADHD difficulties, design for joint use by a parent and young person, use as an ADHD educational tool, use in monitoring ADHD-related difficulties and symptoms, easy to use, possible use to improve daily routines.

Future research is needed on the value of apps for parents of children and young people with ADHD and, in particular, any positive role for apps in the management of ADHD in this age group.

Future research is needed to develop apps in coproduction with key stakeholders and explore the effectiveness of technology-based interventions.

This could result in setting standards for apps for ADHD and other chronic conditions, which could guide users to the optimal apps for their needs.

## Appendix

Multimedia Appendix 1 App summary.

## Abbreviations

ADHD: attention-deficit hyperactivity disorder
ASD: autism spectrum disorder
ASO: app store optimization
F: Female
HCP: health care professional
M: Male
NHS: National Health Service
