# Supplementary material for: ADHD: Is There an App for That? A Suitability Assessment of Apps for the Parents of Children and Young People With ADHD
Source: JMIR Mhealth Uhealth. 2017 Oct 13;5(10):e149. doi: 10.2196/mhealth.7941 (PMC5660294; doi:10.2196/mhealth.7941)
Supplement: Multimedia Appendix 1 [file mhealth_v5i10e149_app1.pdf]

App	Price	Audience	Features	Condition focus	Device(s)	Operating system
App 1	FREE	Parents	Resource for news, features & research	ADHD, OCD, Autism, learning disorders	iPhone, iPad, iPod touch	iOS
App 2	£1.49	Parents, children with ADHD	Step by step directions of daily tasks through songs, monitoring chart, colouring book reward	ADHD	iPhone, iPad, iPod touch	iOS
App 3	FREE	Parents, children with ADHD	Daily audio relaxation recordings	ADD/ ADHD	iPhone, iPad, iPod touch	iOS
App 4	FREE	Parents, 6-8years	Learn to dress by dressing Alex (cartoon character) in order & imitate	ASD, ADHD	iPhone, iPad, iPod touch	iOS
App	FREE	6+,	Story	ADHD	iPad	iOS

5		parents, carers, families, educators	telling about Olly (boy) and Red (ADHD dragon), games			
App 6	FREE	Parents, carers, teachers of kids under 18yrs	Manage medication, plan daily activities, measure treatment results, self-assessment tools, direct Dr communication via sister app	ADHD	Android devices	Android
App 7	FREE	Children/ adolescents with/without ADHD, parents, psychologists, drs, teachers, friends	Test memory in some game levels, dialogue between cartoon characters, links provided to ADHD	ADHD	Android devices	Android

			info			
App 8	FREE	Parents of children with ADHD	Links to ADHD info/resources, sliders to record key times of day, charts, email charts, appt reminders	ADHD	Android devices	Android
App 9	0.99p	Parents of children with various disorders	Click on problem behaviour and find a solution, possible approaches related to special education	ADHD, Autism, Anxiety, behavioural problems	Android devices	Android
App 10	FREE	Parents & special education professionals	Create a visual schedule to support transition times in	Special needs, ASD, developmental delays, genetic syndromes, LDs, ADHD,	Android devices	Android

			day. Illustrate sequence of tasks/sub-steps of a task, can label images with text.	behaviour difficulties. TBI, AD (older adults)		
